# The latissimus dorsi creates a dynamic track for the inferior angle of the scapula during arm abduction in humans

**DOI:** 10.1186/s13018-024-04659-2

**Published:** 2024-03-20

**Authors:** Alp Paksoy, Doruk Akgün, Henry Gebauer, Daniel Karczewski, Lucca Lacheta, John M. Tokish, Aaron Chamberlain, Philipp Moroder

**Affiliations:** 1 Center for Musculoskeletal Surgery, Charité University Hospital, Augustenburger Pl. 1, 13353 Berlin, Germany; 2 University Hospital rechts der Isar, Technical University Munich, German, Germany; 3 Mayo Clinic, Phoenix, AZ USA; 4 Center for Advanced Medicine Orthopedic Surgery Center, St. Louis, MO USA; 5 Washington University and Barnes-Jewish Orthopedic Center, Chesterfield, MO USA; 6 Schulthess Klinik, Zurich, Switzerland

**Keywords:** Scapular dyskinesis, Latissimus scapula overlap, Latissimus-guided track, Latissimus dorsi muscle

## Abstract

**Background:**

The importance of several scapulothoracic muscles, including trapezius and serratus anterior, in maintaining physiological scapula kinematics has been highlighted in the past. However, the relationship between the scapula and the latissimus dorsi muscle remains unclear. Our clinical surgical observation is that the latissimus dorsi does not directly attach but rather runs superficial to the inferior angle of the scapula. Based on this observation, we hypothesise that the latissimus dorsi creates a dynamic track on which the scapula glides under the muscle belly during elevation of the arm, creating the latissimus-scapula overlap (LSO).

**Methods:**

All consecutive patients who had a whole-body computed tomography scan (CT) in case of polytrauma evaluation between 2018 and 2021, with complete depiction of the scapula and latissimus dorsi muscle, were analysed. 150 shoulders in 90 patients with arms up were matched according to their age (within five years), gender, and affected side with 150 shoulders in 88 patients with arms down. Patients with pathologies of the upper extremities or thorax that potentially could alter LSO measurements were excluded. LSO was calculated as a ratio of the measured area of the latissimus dorsi projection on the scapula and the total scapula area.

**Results:**

The mean age of the 178 patients (48 females; 13 males) was 60 years. The arms-up group showed a significantly higher LSO than the arms-down group (19.9 ± 6.3% vs. 2.7 ± 2.2%; *p* < 0.0001). In the arms-up group, approximately one fifth of the scapula was overlapped inferiorly by the muscle belly of the latissimus dorsi, contrary to the almost non-existing LSO in the arms-down group.

**Conclusion:**

With arms up, humans show a significantly higher LSO in comparison to arms down indicating that the latissimus dorsi indeed creates a dynamic track on which the scapula is forced to travel during abduction of the arm. This finding of increased LSO during the elevation of the arm warrants further consideration of the role of the latissimus dorsi in scapula kinematics and potentially scapular dyskinesis.

**Level of evidence:**

Level two diagnostic study.

**Supplementary Information:**

The online version contains supplementary material available at 10.1186/s13018-024-04659-2.

## Introduction

Scapular dyskinesis (SD) is present in as many as 67–100% of athletes with shoulder injuries [1], but also in many asymptomatic individuals [2]. It is characterized by increased protraction with a prominent scapular medial border and an inferior angle, resulting in atypical and inefficient kinematics of the arm and shoulder [3, 4]. Disrupting scapulothoracic kinematics overloads the compensatory musculature, limits shoulder strength and range of motion, and causes pain [5–10]. SD may occur due to several shoulder pathologies, including injury of the acromioclavicular joint, rotator cuff tear, clavicular fracture, shoulder impingement, multidirectional instability, and labral injury [11–18]. Furthermore, scapular muscles are essential contributors to scapular positioning both at rest and during shoulder movement [11, 19]. The upper and lower trapezius muscles, as well as serratus anterior, have been shown to be key muscles for maintaining optimal scapular stability during shoulder motion [20, 21]. While trapezius and serratus anterior muscles initiate upward rotation and posterior tilt, the lower trapezius plays a key role in scapular stability in the overhead position of the arm, as well as in the descent from maximum elevation [22, 23]. The rhomboids, levator scapulae and pectoralis minor assist the trapezius and contribute to controlling medial and lateral scapular translation [23].

The latissimus dorsi originates on the thoracolumbar aponeurosis of T7 through the iliac crest and inserts on the crest of the lesser tuberosity of the humerus with direct and indirect attachments to the inferior border of the scapula in supposedly two-thirds of the cases [24, 25]. It affects scapular motion as the prime mover of the arm and its effect on SD plays a relatively small role in the literature. Recently, a significant relationship between increased latissimus stiffness and altered scapular kinematics due to the pull of the latissimus dorsi on the inferior border of the scapula was found [24]. Thus, regardless of the specific cause of the altered scapular kinematics, an apparent relationship seems to exist between the latissimus dorsi and SD. Furthermore, many anatomy books depict the inferior angle of the scapula as one of the origins of the latissimus dorsi muscle [26–34]. According to our clinical surgical observation, the position of the scapula can change significantly in relation to the muscle belly of the latissimus dorsi, which may create a dynamic track on which the inferior border of the scapula is overlapped by the muscle belly and glides under it during arm movement (Video 1 and 2). This dynamic restraint, dependent on the degree of the latissimus overlap, can theoretically play a significant role in preventing SD by stabilizing the inferior border of the scapula against the chest and optimizing the position of the scapula throughout the range of motion, for example, when performing pull ups. Therefore, the aim of this study was to evaluate the latissimus-scapula overlap (LSO) in patients without any pathologies of the upper extremities or thorax and its variance in arms up and down positions.

## Materials and methods

### Study population

All consecutive patients who underwent whole-body computed tomography (CT) scans for polytrauma evaluation between 2018 and 2021, from our institutional radiology database, with complete depiction of the scapula and latissimus dorsi muscle, were analyzed. A total of 300 shoulders in 178 patients were included in our study, with 150 shoulders in 90 patients having their arms raised (arms-up group) and matched according to age (within five years), gender, and affected side with 150 shoulders in 88 patients with arms in the anatomical position (arms-down group). Patients in the arms-up group were asked to actively raise their arms above their heads by forward flexion and abduction in the anatomical posture, while those in the arms-down group kept their arms in the anatomical position. Both groups were in a prone position during polytrauma evaluation. Patients with pathologies of the upper extremities or thorax that could potentially alter LSO measurements (e.g., fractures, prostheses, dysplasia, or diagnosed soft tissue pathologies such as adhesive capsulitis and rotator cuff disease) were excluded. Approval from the institutional ethics committee was obtained prior to onset of investigation.

### Image measurements

**Fig. 1:**
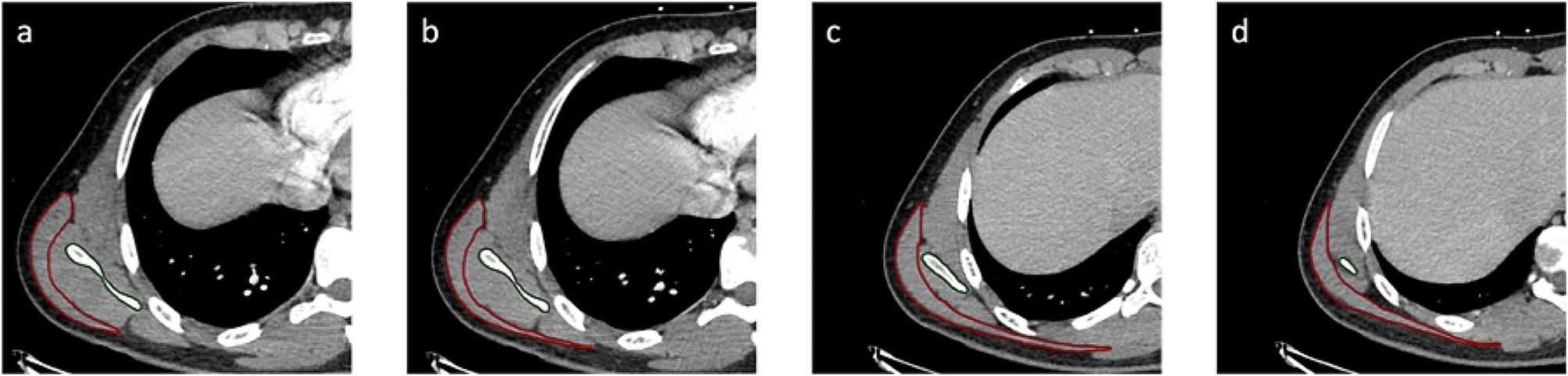
Exemplary axial CT views from cranial to caudal direction (**a**$\to$**d**) of a right shoulder in arms up position. The regions of the scapula covered by the latissimus dorsi muscle are depicted in green, and the borders of the latissimus dorsi muscle in red

**Fig. 2:**
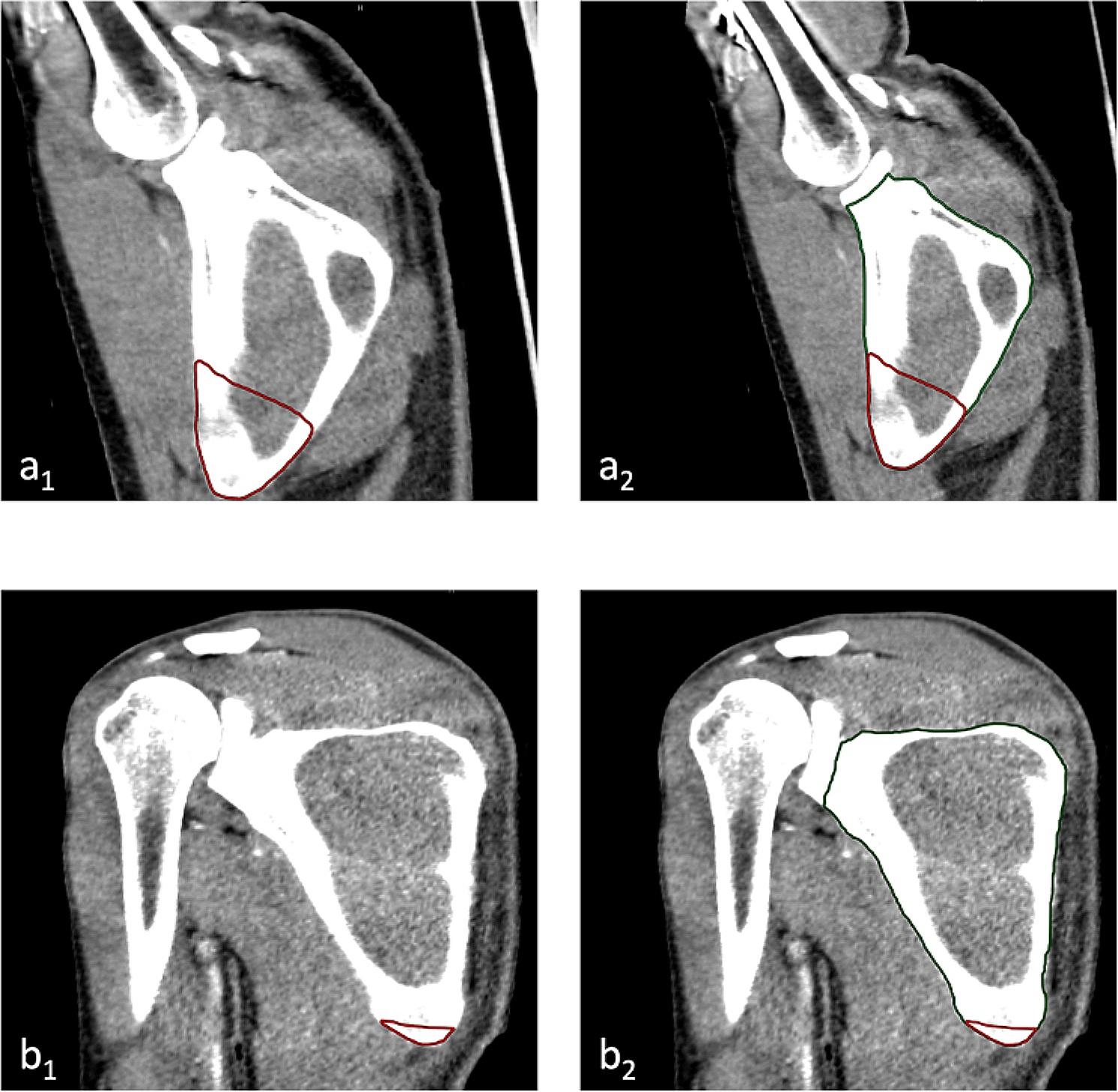
Projection of the latissimus dorsi muscle (red lines) overlapping the scapula in the coronal CT scan of a patient within the arms-up group (**a**_1_) and within the arms-down group (**b**_1_), respectively. The scapula area (green lines) was defined with anatomical borders and a line between the scapular notch and the distal end of the glenoidal cavity as the medio-proximal border (**a**_2_ and **b**_2_)

For all measurements, a standardized axial imaging plane was created using multi-planar reconstruction with the help of Visage software (version 7.1; Visage Imaging, Berlin, Germany). The regions of scapula covered by the latissimus dorsi muscle were manually marked in the axial view (Fig. 1) to generate a segmented model. The projection of this model in the coronal plane was then used to measure the area of the latissimus dorsi projection on the scapula (Fig. 2a_1_ and b_1_). LSO was calculated as a ratio of the measured area of the latissimus dorsi projection against the scapula area, defined with anatomical borders medially and laterally, superiorly with the scapular spine, craniolaterally through a parallel line to glenoid cavity between the scapular notch and the distal end of the glenoid neck (Fig. 2a_2_ and b_2_).

### Statistics

For statistical analysis, IBM SPSS Statistics 25.0 software (IBM, Armonk, NY, USA) was employed. If data were normally distributed, the paired-samples Student’s t-test was used to compare measurement results. If the distribution was abnormal, paired samples were compared using Wilcoxon’s signed rank test. Unpaired samples were analyzed using the Mann–Whitney U test. A p-value < 0.05 was considered significant. To compare LSO measurements among groups, the one-way ANOVA test was used.

## Results

**Fig. 3:**
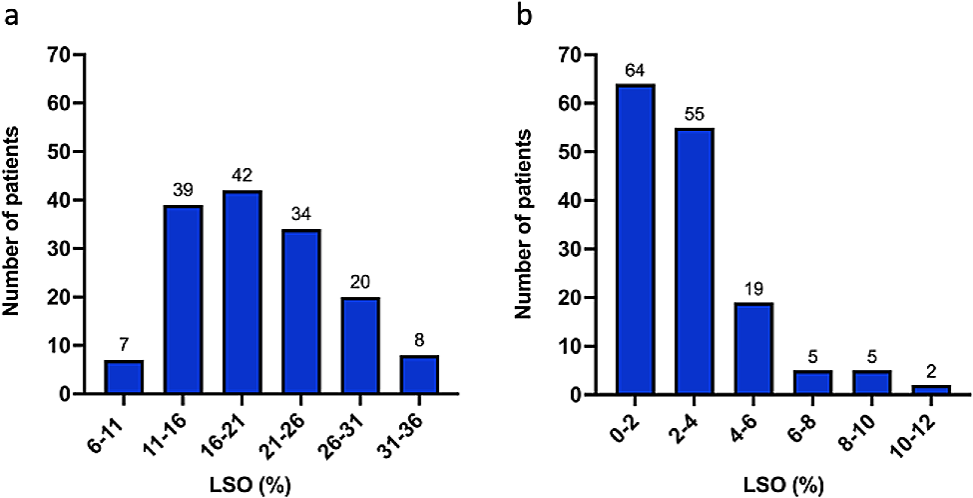
Frequency of LSO percentages within the arms-up (**a**) and -down (**b**) group was illustrated in 6% intervals

**Fig. 4:**
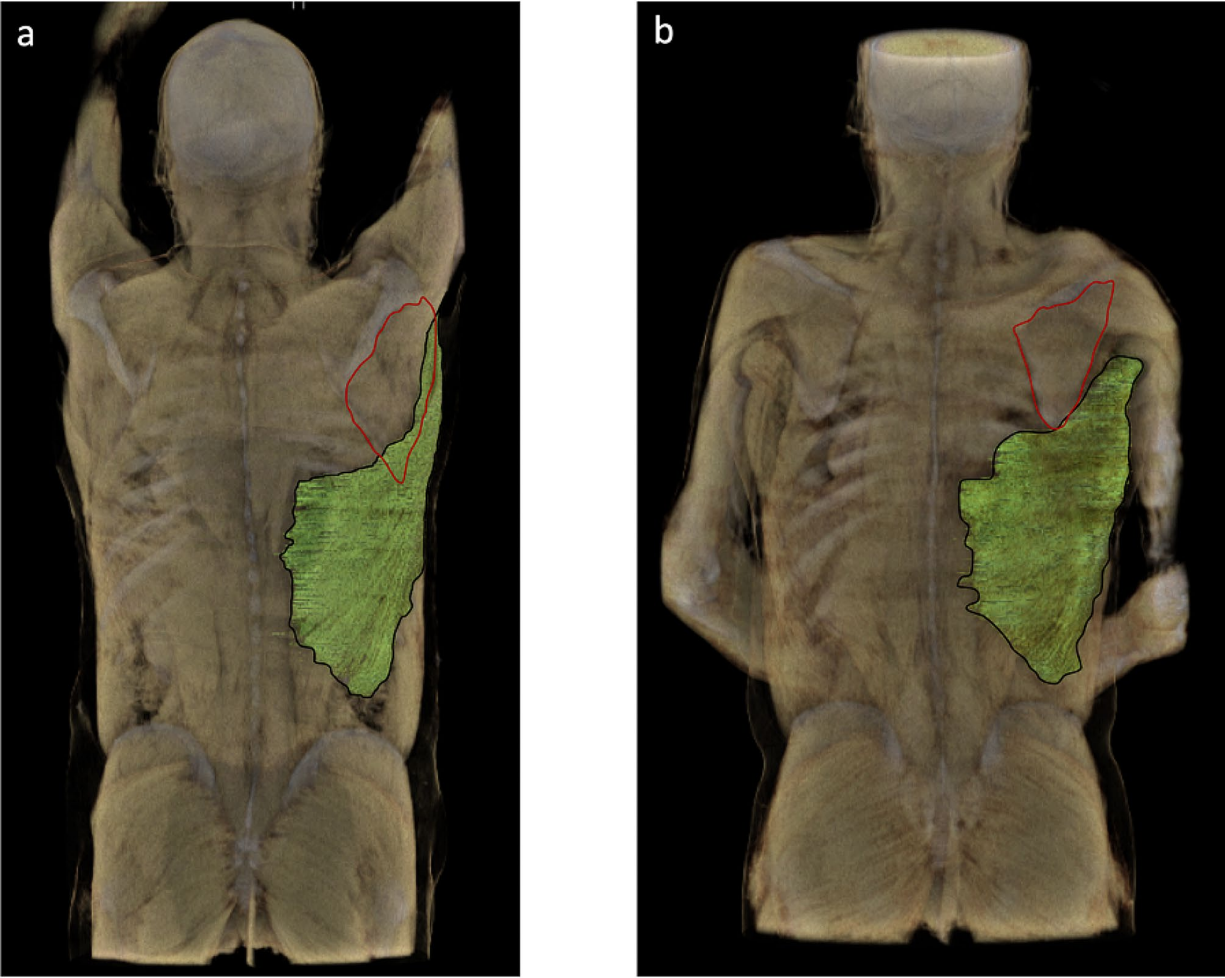
Projection of the latissimus dorsi muscle (depicted in green) overlapping the scapula (its measured area inferior to the scapular spine marked off in red) in 3D segmentation of a patient within the arms-up group (**a**; LSO = 24.8%) and within the arms-down group (**b**; LSO = 2.5%), respectively

The study cohort comprised 48 female and 130 male patients with a mean age of 60 years (range, 20–91 years) and without any pathologies of the upper extremities or thorax. The arms-up group consisted of 24 female and 66 male patients, while the arms-down group had 24 female and 64 male patients (Table 1). LSO showed a significant difference between the arms-up and -down groups, with significantly higher LSO in arms-up group compared to the arms-down group (19.9% $\pm$ 6.3% vs. 2.7% $\pm$ 2.2%; *p* < 0.0001) (Fig. 3). (Table 1). In order to visualize this significant difference, three dimensional (3D) segmentation was conducted for the arms-up and -down positions (Fig. 4). While there was a trend in mean LSOs between female and male patients in the arms-up group, with higher LSO in men (*p* = 0.08), the difference in mean LSOs between sexes in the arms-down group was significant, with lesser LSO in men (*p* = 0.04). No significant difference in LSO was detected between the age groups (Table 1) within the arms-up and -down groups. The mean LSO in the arms-up group changed significantly according to the side (right: 21.2 $\pm$ 6.1% versus left: 18.7 $\pm$ 6.2%; *p* = 0.02), whereas in the arms-down group, no significant difference was identified between the sides.

**Table 1:** Comparison between the arms-up and -down groups according to their age, gender and injured side

	Arms-up	Arms-down	*p*-value
Total shoulders, n	150	150	-
Patients, n (female : male)	90 (24 : 66)	88 (24 : 64)	-
Mean age in years	60 $\pm$ 20.5	59 $\pm$ 20.4	0.30
Mean LSO in %	19.9 $\pm$ 6.3	2.7 $\pm$ 2.2	**< 0.0001**
Mean LSO in % according to sex (female : male)	18.4 $\pm$ 4.5 : 20.5 $\pm$ 6.7 (*p* = 0.08)	3.3 $\pm$ 3.1 : 2.5 $\pm$ 1.8 (***p*** = **0.04**)	**< 0.0001** **< 0.0001**
Mean LSO in % according to age			
20–39	22.0 $\pm$ 5.7	2.6 $\pm$ 2.0	**< 0.0001**
40–59	19.2 $\pm$ 7.3	2.7 $\pm$ 2.3	**< 0.0001**
60–79	19.4 $\pm$ 5.9	2.6 $\pm$ 2.3	**< 0.0001**
> 80	19.6 $\pm$ 5.9	3.2 $\pm$ 2.2	**< 0.0001**
Mean LSO in % according to side (right : left)	21.2 $\pm$ 6.1 : 18.7 $\pm$ 6.2 (***p*** = **0.018**)	2.9 $\pm$ 2.3 : 2.6 $\pm$ 2.1 (*p* = 0.63)	**< 0.0001** **< 0.0001**

## Discussion

The primary objective of this study was to assess the variation in LSO in patients with arms up and down and without any pathologies of the upper extremities or thorax. Our data revealed a significant difference in mean LSO between the arms-up and -down groups, with the arms-up group exhibiting a notably higher LSO than the arms-down group. The observed variance based on arm position led us to question the anatomical relationship between the latissimus dorsi muscle and the scapula.

Literature suggests a potential attachment of the latissimus dorsi muscle to the inferior angle of the scapula [26–34]. To our knowledge, the studies by Williams et al. [35] and by Pouliart et al. [25] are the only ones which describe a possible insertion of the latissimus dorsi to the inferior angle of the scapula. Pouliart et al. [25] studied 100 cadaver specimens and identified three possible types for the relationship between the latissimus dorsi and the inferior angle of the scapula: In 43 out of 100 specimens, they observed muscular fibers of the latissimus dorsi emerging from the inferior angle of the scapula (type 1, scapular connection), in 36 out of 100 specimens few fibrous strands between the two (type 2a, indirect attachment) and in 21 out of 100 specimens a bursa and no connective tissue (type 2b, no attachment). This firm attachment of the latissimus dorsi to the inferior angle of the scapula could not be explained under the light of our results, since our LSO measurements illustrated a significant difference between arms up and down groups. In parallel to that, our 3D segmentations (Fig. 4) and cadaver illustrations (Video 1 and 2) supported our radiologic measurements and the concept of a “latissimus guided track”, illustrating the inferior border of the scapula traveling dynamically under the muscle during arm movement. This dynamic restraint illustrates a significant increase in LSO during arm abduction and since the latissimus tendon inserts on the humerus, the position of this track changes with the position of the arm, presumably optimizing the position of the scapula throughout the range of motion. This dynamic track may stabilize the inferior border of the scapula against the chest and could be a factor in deciphering the pathophysiology of SD.

The upper and lower trapezius muscles along with the serratus anterior muscles have been shown to be the greatest contributors to scapular stability and mobility [20, 21, 36], playing a crucial role in explaining primary and secondary SD. In comparison to trapezius and serratus anterior muscles, the role of latissimus dorsi muscle in the pathomechanism of SD remains less explored. In a cross-sectional study with 19 collegiate swimmers, Laudner et al. measured latissimus dorsi stiffness of the dominant arm while in a lengthened position with a myotonometer and used an electromagnetic tracking device to measure scapular kinematics at humeral elevation angles of 30°, 60°, 90° and 110° within the scapular plane [24]. They illustrated moderate-to-good relationships between increased latissimus dorsi stiffness and increased scapular upward rotation and posterior tilt, as well as decreased scapular internal rotation. These alterations in positioning and motion of the scapula were attributed to the pull of the latissimus dorsi on the inferior border of the scapula and early scapular elevation during arm elevation, resulting in a noticeable disruption in scapula–humeral rhythm. Our cadaver studies and radiologic measurements support this relationship, suggesting that a contracted latissimus dorsi could potentially place increased pressure with its dynamic track throughout the range of motion on the inferior scapular border moving it anteriorly towards the thorax resulting in increased posterior tilt [24]. Regardless of the specific explanation of the altered scapular mechanics, a distinct relationship does appear to exist between latissimus dorsi muscle and scapular kinematics.

This study has some limitations. First, LSO measurements were conducted in a cohort without any pathologies of the upper extremities or thorax and thus the results cannot explain a pathologic situation. Second, the points measured were the starting and ending points of the tract, providing static data for explaining a dynamic phenomenon. Further studies employing 3D measurements of scapular motion during arm elevation are recommended for instance with magnetic resonance imaging or new motion analysis techniques optimized for thoracoscapular movement to identify this dynamic track. Third, the changes in muscle parameters during active motion like activity, strength, tightness and degree of contraction could provide a more comprehensive understanding and strengthen the radiological and anatomical observance presented in this study. Fourth, CT scan allows for bony visualization and not the optimal method for precise description of the latissimus dorsi fibers and their pathway.

## Conclusion

Patients with arms up showed a significantly higher LSO compared to patients with arms down. The arm position-dependent change in LSO may contribute to understanding altered scapular kinematics in SD. Further research is required to explore the precise mechanisms of the “latissimus guided track” in patients with SD and its potential implications for treatment interventions.

### Electronic supplementary material

Below is the link to the electronic supplementary material.

**Video 1**: Increase in LSO while arms reaching the arms-up position and its decrease during arm adduction, illustrating a dynamic track guided by the latissimus dorsi muscle throughout the range of motion

**Video 2**: “Latissimus guided track” after the excision of the fascia for better illustrative purposes

## Data Availability

No datasets were generated or analysed during the current study.

## Abbreviations

CT: Computed tomography
LSO: Latissimus-scapula overlap
SD: Scapular dyskinesis
3D: Three-dimensional
